# Computational Modeling of 3D Tumor Growth and Angiogenesis for Chemotherapy Evaluation

**DOI:** 10.1371/journal.pone.0083962

**Published:** 2014-01-03

**Authors:** Lei Tang, Anne L. van de Ven, Dongmin Guo, Vivi Andasari, Vittorio Cristini, King C. Li, Xiaobo Zhou

**Affiliations:** 1 Department of Translational Imaging, The Methodist Hospital Research Institute, Houston, Texas, United States of America; 2 Department of Radiology, Wake Forest School of Medicine, Winston-Salem, North Carolina, United States of America; 3 Department of Physics, Northeastern University, Boston, Massachusetts, United States of America; 4 Department of Pathology, Cancer Research and Treatment Center, Department of Chemical and Nuclear Engineering, and Center for Biomedical Engineering, The University of New Mexico, Albuquerque, New Mexico, United States of America; University of Catania, Italy

## Abstract

Solid tumors develop abnormally at spatial and temporal scales, giving rise to biophysical barriers that impact anti-tumor chemotherapy. This may increase the expenditure and time for conventional drug pharmacokinetic and pharmacodynamic studies. In order to facilitate drug discovery, we propose a mathematical model that couples three-dimensional tumor growth and angiogenesis to simulate tumor progression for chemotherapy evaluation. This application-oriented model incorporates complex dynamical processes including cell- and vascular-mediated interstitial pressure, mass transport, angiogenesis, cell proliferation, and vessel maturation to model tumor progression through multiple stages including tumor initiation, avascular growth, and transition from avascular to vascular growth. Compared to pure mechanistic models, the proposed empirical methods are not only easy to conduct but can provide realistic predictions and calculations. A series of computational simulations were conducted to demonstrate the advantages of the proposed comprehensive model. The computational simulation results suggest that solid tumor geometry is related to the interstitial pressure, such that tumors with high interstitial pressure are more likely to develop dendritic structures than those with low interstitial pressure.

## Introduction

Tumors have highly irregular properties compared to normal tissue, including multiple cell phenotypes, heterogeneous density, high intra-tumoral pressure, and tortuous vasculature [1]–[4]. The complexity of tumor morphology can cause the use of conventional methods for anti-tumor drugs become inefficient and expensive. Nowadays, mathematical models based on underlying biological properties can provide a powerful tool to facilitate drug development and pre-clinical evaluation. Such models consist of realistic quantitative mathematical descriptions of biological phenomena that can be calibrated by comparison with experimental data. The primary advantage of mathematical modeling is its controllable characteristics and high efficiency compared to laboratory experiments. Underlying biological mechanisms may be revealed by comparing the computational simulation results with experimental observations. Moreover, by simply changing parameter values of the descriptive equations, the significance and functions of variables representing specific biological features can easily be tested.

It has been well established that solid tumors grow in two distinct phases: the initial growth being referred to as the avascular phase and the later growth as the vascular phase. The transition of solid tumor growth from the relatively harmless avascular phase to the invasive and malignant vascular phase depends upon the crucial process of angiogenesis. Tumor angiogenesis has been studied since 1960s and it is a physiological process in which the tumor cells secrete diffusible substances known as Tumor Angiogenesis Factor (TAF) into the surrounding tissue to stimulate the formation of new capillary blood vessels. The new blood vessels grow towards and penetrate the tumor; they are crucial for supplying the tumor cells with vital nutrients and disposing of waste products. Araujo and McElwain presented a comprehensive review on the history of mathematical modeling of solid tumor growth [5] and for the discussion and history on angiogenesis discoveries, see the review paper by Kerbel [6].

In the past decades, many studies have been dedicated to the modeling of tumor growth and treatment, theoretically and computationally. Various mathematical approaches and techniques have been used to model tumor growth from many aspects and view points. Avascular tumor models by [7]–[9] were proposed to simulate tumor growth based on basic mechanisms. Multi-dimensional avascular tumor growth models [10], [11] have been utilized to perform more detailed simulations; however, such models are limited by a lack of realistic vasculature and associated transport phenomena. Therefore, as new discoveries on tumor-induced angiogenesis were reported and with advances in computing technology, vascularized tumor models have become increasingly prevalent in recent years [12]–[16]. For example, Sinek et al. [17] developed a two-dimensional model for tumor growth and angiogenesis modeling and applied the model to predict nanoparticle chemotherapy. Sanga et al. [18] extended the model to three dimensions to better recapitulate heterogeneity of tumors for the study of drug treatment. This model treated tumor cells as a single population and did not incorporate drug concentration-dependent cell cycle heterogeneities. Tumor growth with angiogenesis have also been modeled using multicell [19] and multiscale [20], [21] techniques, as well as multiscale modeling by incorporating drug therapy at the extracellular level [22] and drug combination for tumor treatment [23]. Nevertheless, existing models lack of sufficient resolution to evaluate cancer drug effects at the cellular level. Advanced mathematical models incorporating more details are needed to provide further insights into the tumor progression and response to chemotherapy.

In this paper, we present a three-dimensional mathematical model of tumor growth coupled with tumor-induced angiogenesis, in which we use empirical methods to simulate experimental observations. This enables us to make realistic predictions of the transition from avascular to vascular tumor while avoiding getting stuck in obscure mechanisms or complex calculations. Several new methods and concepts in modeling are introduced: Firstly, we propose an efficient method for calculating intra-tumoral interstitial pressure that incorporates the contributions of both tumor cells and endothelial cells. Secondly, we introduce a concept called the “Virtual Branching Hotpoint (VBH)” to regulate the vessel branching process. Both the vessel growth rate and appearance of VBH are calculated based on empirical formulas that describe the local concentration of tumor angiogenesis factor (TAF). Thirdly, cell proliferation is assumed to be controlled by “Cell Vital Energy (CVE)”, a new concept describing cell progression towards division according to the local microenvironment, nutrient (oxygen) availability and waste product (carbon dioxide) disposal, and drug concentration. And fourthly, drug pharmacodynamics is modeled empirically to predict the overall therapeutic effects upon tumor cells. The dynamics of oxygen, carbon dioxide, TAF, and drug are modeled using partial differential equations. We envision that the proposed model will serve as a simulation platform for tumor growth prediction and chemotherapy evaluation. We also performed a series of computational simulations to shed light on tumor progression in the three dimensional space and provide detailed insights into tumor response to various drugs. The simulation results suggest that solid tumor geometry is related to interstitial pressure, such that tumors with high interstitial pressure are more likely to develop dendritic structure compared to those with low interstitial pressure.

## Mathematical Model and Methods

**Figure 1:**
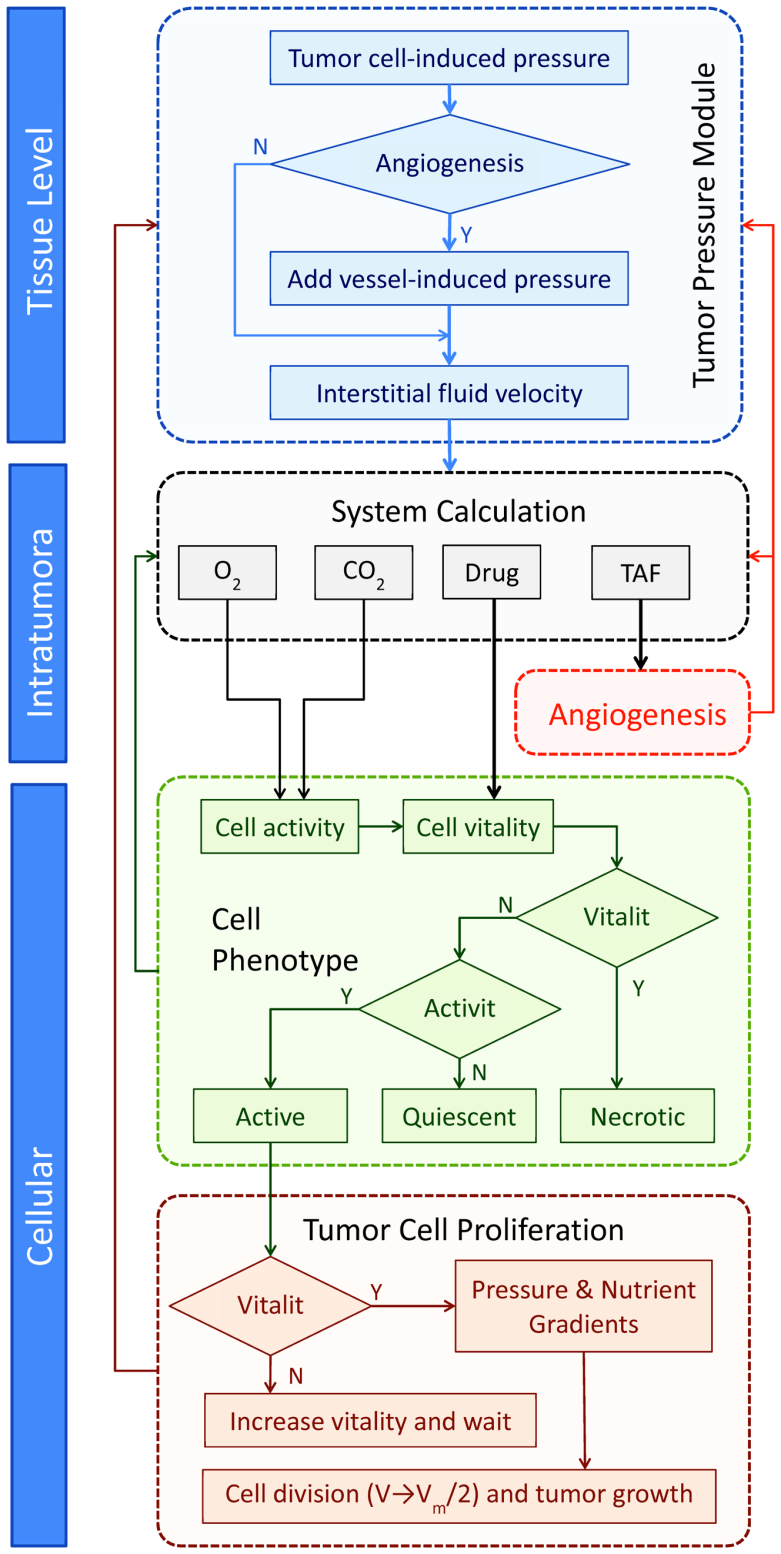
Multi-scale modeling system structure.

Multiscale 3D tumor modeling is utilized to detail mechanisms of tumor growth. Pressure in the avascular tumor state has been implicated to interstitial fluid compression by densely packed, rapidly dividing tumor cells [24]–[26], which we term here cell-induced tumor pressure (CTP). Upon neovascularization, vessel perfusion was also assumed to play a role in generating the so-called vasculature-induced tumor pressure (VTP). A comprehensive growth pressurization model (GPM) is developed to adaptively calculate tumor pressure at the tissue-level as a function of both CTP and VTP. Within tumor tissue, gradients of interstitial pressure have been studied to impact the transport of macromolecules such as metabolites, growth factors, and drugs [27], [28]. To scale the model to the level of heterogeneities in mass transport, we built a series of mass conservation equations based on biophysical and biomechanical rules. Specifically, we considered oxygen (*O*_2_) and carbon dioxide (*CO*_2_) as the primary metabolites that regulate cell activity, and tumor angiogenesis factor (TAF), an angiogenic growth factor capable of altering tumor pressure and growth factor distribution. At the cellular level, cell phenotype was determined by cell activity and cell vital energy (CVE). System equations describing drug distribution directly influenced the CVE, such that individual cells could be defined as active, quiescent, or necrotic. Active tumor cells were assumed to divide when the CVE reached a specific threshold. The resulting changes in cell number and phenotype were fed back into the tumor pressure and mass transport calculations. The overall structure of this model is summarized in Fig. 1. All parameters were defined in such a way that calibration of individual variables and tuning of separate modules to experimental observations would be able to effectively simulate tumor progression from an avascular to vascular state in the presence and absence of different drugs.

### 2.1 Solid Tumor Growth Model

Many biophysical and biochemical factors that play important roles in tumor growth, including tumor interstitial pressure, tissue density, nutrient availability, and metabolic waste clearance have been widely investigated in previous tumor modeling studies [13], [18], [29]. In this work, we treat tumor pressure and metabolite concentrations as representative governing factors of both avascular and vascular solid tumors. Tumor interstitial pressure has been implicated in cell proliferation [24]–[26] and migration outwards towards regions of lower pressure. Tissue oxygen and carbon dioxide levels are assumed to regulate tumor cell activities including oxygen consumption rate, carbon dioxide secretion rate, and drug uptake rate during chemotherapy.

High interstitial pressure is thought to be generated via various mechanisms including intracellular forces, interstitial fluid compression, and blood vessel perfusion. Yet, none of these mechanisms is considered to be dominant and instead are thought to work together [27]. We therefore propose a new tumor pressure calculation method called GPM describing tumor pressure as a combination of pressure caused by cell-induced tumor pressure (CTP) and vascular perfusion-induced tumor pressure (VTP). Firstly, avascular tumor pressure can be calculated as a sum of pressure perceived by tumor cell *X*_0_ at a grid point (*x*_0_, *y*_0_, *z*_0_) due to pressure caused by tumor cell *X_i_* located at (*x_i_*, *y_i_*, *z_i_*): 
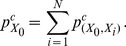
(1)


The pressure can be expressed in the form of Gaussian-like function: 
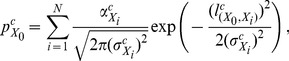
(2)where 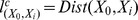 is the Euclidean distance between tumor cell *X_i_* and tumor *X*_0_ at the reference point (*x*_0_, *y*_0_, *z*_0_). Because tumor pressure is related to tumor cell density, we define the parameters 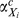 and 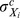 as 
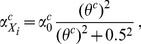
(3a)

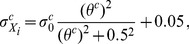
(3b)

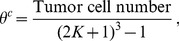
(3c)describing the parameters' dependency on cell density *θ^c^*, which is defined as tumor cell number fraction of total neighboring cells within the *K* neighboring cells of cell at *X*_0_. Tumor cell number *N* = (2*K*+1)^3^ is the total grid points within *X*_0_-centered *K*-neighboring cubic with a length of (2*K*+1) in each dimensional space. In areas of high tumor cell density, compact intracellular space and compressed interstitial fluid result in higher pressure compared to normal tissue or peripheral regions of solid tumor where cell density is lower.

For vascularized tumors, perfusion into the solid tumor contributes to tumor cell pressure, which is calculated similarly to tumor cell induced pressure, denoted as 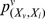. This pressure is caused between certain endothelial cell at 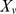 and its neighboring grids *X_i_*. We calculated 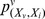 for each endothelial cell within *K*-neighboring cubic with length of (2*K*+1), and then added VTP to CTP to get the final combined tumor pressure *p* in vascularized tumor. We define 
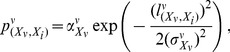
(4a)

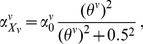
(4b)

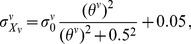
(4c)where 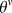 is calculated similar to Eq. 3c. Following [30], Darcy's law for flow through a porous medium can be used to describe interstitial fluid transport in the tumor, generally given as 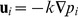
(5)where *k* the is hydraulic conductivity of the interstitium and *p_i_* is the interstitial pressure.

It has been well studied that enhanced proliferation of tumor cells consequently increases the demand for nutrients that are necessary for the synthesis of macromolecules (DNA, RNA, proteins) and important as the carbon source for generation of metabolic energy in tumor cells. Nutrients such as glucose, amino acids, fatty acids, vitamins, and micronutrients are hydrophilic. They do not easily permeate across cell's plasma membrane, particularly mammalian cells. They also require specific transporters for their uptake by cells [31]. Therefore, for the sake of simplicity, in our model we use only oxygen for generic nutrient concentration. Oxygen (*O*_2_) is a crucial element in the body which is needed by cells for aerobic metabolism. The availability of oxygen plays an important role in a way that it affects tumor cell metabolism, angiogenesis, growth, and metastasis [32], [33]. Oxygen, which is bound to hemoglobin in red blood cells, is carried and delivered by blood vessels to every part of our body. There are three varieties of blood vessels: arteries, capillaries, and veins. The arteries carry the oxygen-rich blood away from the heart to the capillaries, at which the oxygen is released and rapidly diffuses into the tumor tissue. The tissue also releases its waste products such as carbon dioxide (*CO*_2_) that passes through the wall of capillaries and into the red blood cells, which is transported by the veins back to the lungs and heart. Hence, the spatio-temporal evolution for oxygen concentration (*n*) is assumed to occur through diffusion, production by blood cells, removal by tumor cells, and convection of the interstitial fluid flow: 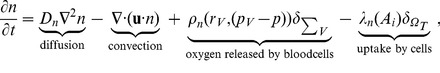
(6)where *D_n_* is oxygen diffusion coefficient in the tumor tissue, the third term 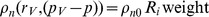
(7)denotes the kinetics of oxygen supply by blood cells (the process is occurred in neo-vessels and denoted by 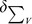, 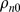 is the rate of oxygen supply, *R_i_* is the radius of blood vessel defined in Eq. 20, and 
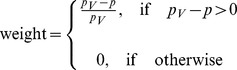
is the pressure gradient through vessel wall, 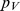 is vascular pressure. Oxygen is assumed to decay with each tumor cell activity *A_i_* (refer to Eq. 12). The process is occurred in cells (denoted by 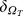) and is expressed as 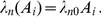
(8)


The parameters 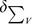 and 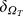 are indication functions for neo-vessels and tumor cells, respectively and remain as such for all equations in this paper.

Tumor cells not only consume large amounts of oxygen and nutrients, but also accumulate metabolic wastes during tumor growth process due to inefficient drainage. This can result in reduced cell activity and biosynthesis or even cell necrosis. In this paper, we consider carbon dioxide (*CO*_2_) as a representative waste metabolite in order to study its influence upon tumor growth. The rate of change of carbon dioxide (*w*) spatially and temporally is assumed to occur through diffusion, convection, production by cells, and removal by red blood cells in the blood vessels (or capillaries), hence 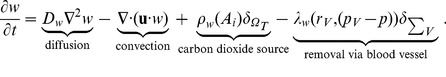
(9)


The carbon dioxide secretion rate 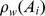 is assumed to be proportional to cell activity *A_i_* and given by 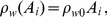
(10)where tumor cell activity *A_i_* is defined in Eq. 12, and the carbon dioxide residue is taken up by blood vessels, expressed as 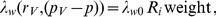
(11)where 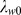 is the rate of carbon dioxide consumption by blood vessels, and *R_i_* and weight are previously defined. Low *CO*_2_ levels can increase cell activity, maintain a high cell proliferation rate, high oxygen consumption rate, and carbon dioxide secretion rate.

Cell activity of each cell *A_i_* plays an important role in defining cell cycle and is influenced by cell metabolism, protein/DNA synthesis, ligand binding, etc. Here the expression is dependent on the concentration of oxygen (*n*) and carbon dioxide (*w*), given by 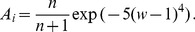
(12)


We propose a new concept called “Cell Vital Energy (CVE)” denoting the energy stored within a given cell for proliferation. We assume that a tumor cell adds CVE according to the following equation 
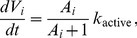
(13)where *k*_active_ is a constant. If the CVE depletes (*A_i_*≤0), the cell is considered to be dead; on the contrary, if the CVE reaches a proliferation threshold, the cell begins to divide into two daughter cells. *A_i_* = 0.5 is set as the threshold for separating active tumor cells and quiescent tumor cells. When activity is above 0.5, cells actively synthesize proteins, add CVE for proliferation, and consume CVE at certain rate according to cell activity, which is depicted by the Hill function in Eq. 13. When activity falls below 0.5, tumor cells become quiescent and stop adding vital energy for proliferation. At the same time, they consume vitality at very low rate *k*_quiescent_ because of normal housekeeping activities. Therefore, the rate of tumor cell vital energy consumption defined as 
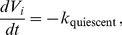
(14)and *k*_quiescent_ is a constant.

**Figure 2:**
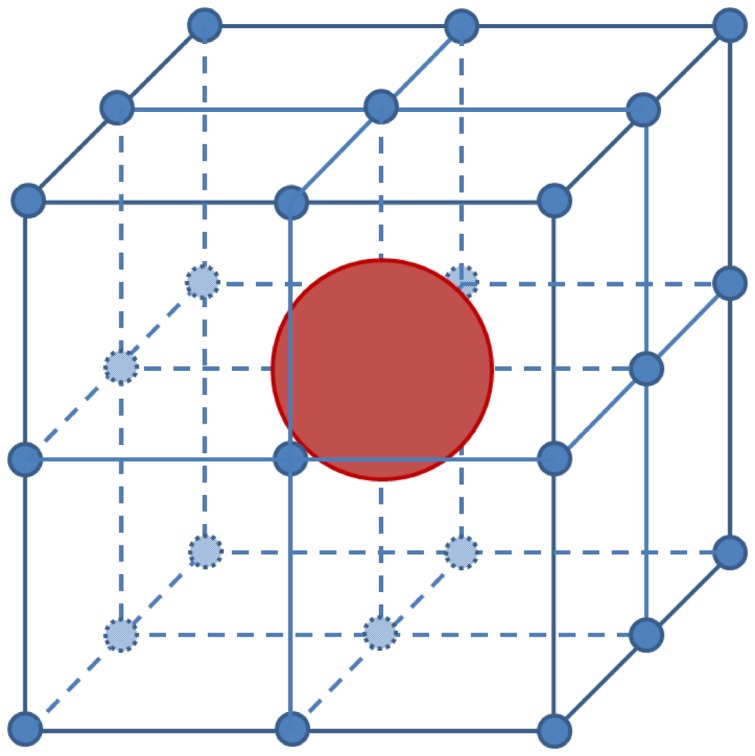
An illustration showing one cell located in the center of cube surrounded by 26 neighboring grid points. The potential directions for spatial transition are calculated according to pressure gradients along these 26 directions.

Tumors grow mainly through uncontrollable cell proliferation [34]. In our model, we treat each cell as located in the center of a three-dimensional cube surrounded by 26 neighboring grid points, as shown in Fig. 2. During cell division, potential directions for spatial transition are calculated according to pressure gradients along these 26 directions. Tumor cells are assumed to migrate or proliferate towards directions of lower interstitial pressure, and as a result, dividing cells inside the solid tumor successively push other cells towards peripheral region. Cell spatial transition probabilities are calculated according to this method of space availability or density dependency, where space with low cell density indicates higher transition probability. We determine areas with low cell density or grids that are not occupied by cells, then we calculate pressure gradient between the dividing cell and free grids. The dividing cell will move towards the grid with highest pressure gradient.

### 2.2 Solid Tumor Angiogenesis Model

Tumor cells consume nutrients more rapidly than normal cells. This leads to hypoxia in the center of avascular tumors once a threshold volume is reached. In response to hypoxia, tumor cells begin to secret tumor angiogenesis factor or TAF to induce new vessels to sprout from pre-existing vasculature towards hypoxia regions [35]. In this paper, tumor vessel growth is determined by TAF density as well as tumor interstitial pressure, under the assumption that TAF secretion rates are higher under hypoxic conditions. TAF concentration field is described by the following partial differential equation 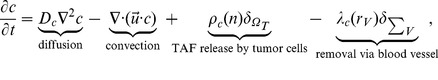
(15)where cells' TAF secretion rate 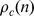 is assumed to be proportional to their oxygen level and given by 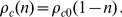
(16)


Similar to metabolic waste removal, the rate of TAF removal drops in high pressure regions, hence 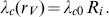
(17)


Tumor-induced blood vessels are assumed to grow towards dense areas of TAF. The probability of endothelial cell migration was considered in six directions, denoted as directional derivative at spatial grid (*x_i_*, *y_i_*, *z_i_*). According to [36], blood pressure is assumed to be highly related to vessel growth rate, especially the pressure difference between inside and outside of tip vessels. High pressure differences propel tip endothelial cells to rapidly proliferate. The endothelial cell proliferation cycle-pressure relationship is given by 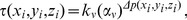
(18)where parameters 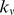 and 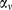 can be calibrated according to measured normal vessel growth rate, and 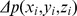 is the pressure difference across vessel wall from inside to outside.

**Figure 3:**
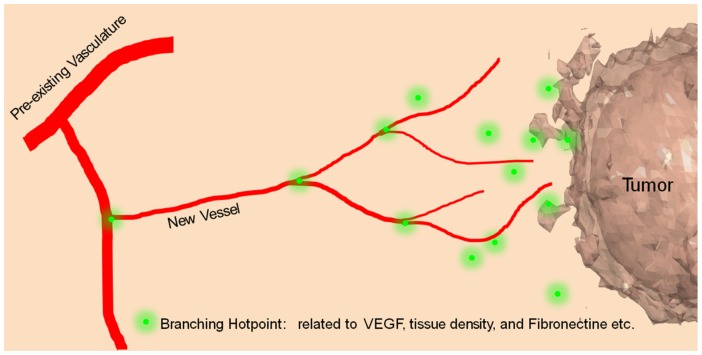
Branching Hotpoint (BH)-induced tumor vasculature branching. Green dots indicate area of enhanced concentration of stimuli for vessel branches.

High resolution images of tumor vasculature show that tumor vessels are highly tortuous [37] and the branching pattern is greatly different from normal blood vessel networks. To simulate the aberrant vasculature, we propose a new method for calculating tumor vasculature branching which we define “Branching Hotpoints” (BHs), located in the tissue at which vessels branch out, as shown in Fig. 3. BHs are related to TAF concentration, tissue density, and fibronectin density. In our model, we only consider TAF for simplicity. We calculate the possibility of BH for each grid point following similar approach as Eq. 18, 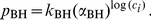
(19)where *k*_BH_ and *α*_BH_ are constants. The distribution of BHs is recalculated during tumor progression. We assume that TAF-induced vessels cannot grow into tumor necrotic core due to adverse pressure and cell density. Therefore, as TAF concentration, tissue density, and interstitial pressure arise at the tumor center, the angiogenic vasculature becomes denser and much more tortuous. Vessel age, as well as vessel radius, continue to grow with each iteration after sprouting from pre-existing vessels, and as a result, tumor vessels vary spatially and temporally in their ability to deliver nutrients and remove wastes. Vessel maturation provides nutrients to starving tumor cells, thereby stimulating further tumor growth and eventually leading to metastasis (metastasis is beyond the focus of this paper). In this way, tumor growth and angiogenesis are modeled as coupled processes.

### 2.3 Drug Treatment

Chemotherapy is an important anticancer approach for the treatment of both primacy tumors and distant metastases. Generally, these drugs can be categorized into two main types: anti-angiogenic drugs and cytotoxic drugs. Anti-angiogenic drugs such as bevacizumab, for example, inhibit the growth of new vasculature by lowering the TAF secretion rate, deactivating TAF, preventing TAF-mediated signaling, or inducing endothelial cell apoptosis directly. Cytotoxic drugs such as Cisplatin, for example, induce damage to tumor cell DNA in order to prevent cell replication. In our model we apply cytotoxic drugs that act directly at the tumor cell level. Vessel radius can be calculated based on endothelial cell age Age*_i_*, 
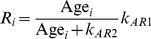
(20)where *R_i_* is vessel radius calculated from Age*_i_*, *k_AR_*_1_ and *k_AR_*_2_ are constants. Vessels are pruned when Age*_i_* = 0.

In order to study the pharmacodynamics of both treatment types, we define the drug distribution inside tumor microenvironment as follows, 
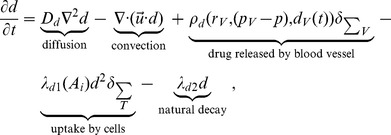
(21)where *D_d_* is drug diffusion coefficient in tumor tissue, 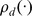 is drug supply rate through new vessels and following [30] is defined as 
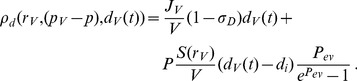
(22)


In the first term of the right hand side of Eq. 22 above, 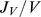 is drug's volumetric flow rate out of the vasculature per unit volume of tissue and given by 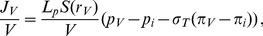
(23)where *L_p_* is the hydraulic conductivity of tumor microvascular wall, 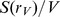 is the surface area per unit volume for drug transport in the tumor, 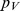 is the vascular pressure, 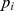 is the interstitial pressure, 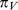 is the osmotic pressure of the drug, 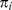 is the osmotic pressure of the interstitium, and 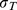 is the average osmotic reflection coefficient for drug. In the second term of the right hand side of Eq. 22, the Peclet number 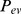 is the ratio of convection to diffusion magnitude across tumor capillary wall and given by 
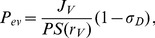
(24)where 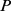 is the vascular permeability coefficient and *d_i_* is the interstitial drug concentration. In both terms, 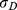 is the osmotic reflection coefficient (different value than 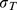) and 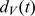, which has been modeled and used in [22], is the time course drug concentration within blood inside tumor capillary that comes from the predictions of organ level compartmental drug delivery or direct measurements. In this paper, we set 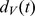 to constant 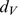. In the Eq. 21, the drug consumption by tumor cells is expressed as 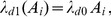
(25)where it is linear with cell activity 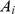, and 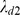 is the rate of drug natural decay.

We extend Eq. 13 by replacing 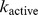 as 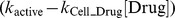 to depict the effects of drug treatment at the cellular level. If cell vital energy drops below zero, the cell is considered as apoptotic in our model, which is an irreversible transition from quiescent status. When cell volume *V_i_* reaches mitosis threshold, the tumor cell begins to divide to two daughter cells with half of original tumor vital energy, which is proposed based on basic cell mitosis theory. By this means, we are able to study dynamic effects of different drugs upon tumor cells.

## Computational Simulation Results

Small tumors and metastases, on the order of several millimeters or less, cannot be detected by modern imaging techniques. Fortunately, chemotherapy can be utilized to suppress the development of small tumors. Using Matlab®, we performed a series of computational simulations to simulate this tumor growth and its corresponding treatment response. All parameter values used in the computational simulations are listed in Table 1, unless specified otherwise. Source code of our model can be found at http://www.wakehealth.edu/CTSB/Software/Software.htm

**Table 1:** Summary of parameter values for 3D tumor model and angiogenesis.

Symbol	Value	Unit	Description	Reference
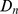	8×10^−14^	*m* ^2^/s	Oxygen diffusion coefficient	[29]
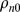	6.8×10^−4^	*mol/*(*m* ^3^ *s*)	Oxygen supply rate	[29]
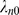	3×10^−5^	*ml/*(*cm* ^3^ *s*)	Oxygen consumption rate	[53]
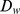	4×10^−14^	*m* ^2^/*s*	Carbon dioxide diffusion coefficient	Estimated
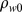	1×10^−5^	*mol/*(*m* ^3^ *s*)	Carbon dioxide secretion rate	Estimated
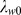	2.5×10^−5^	*ml/*(*cm* ^3^ *s*)	Carbon dioxide consumption rate	Estimated
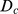	1.2×10^−13^	*m* ^2^/*s*	TAF diffusion coefficient	Estimated
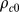	2×10^−9^	*mol/*(*m* ^3^ *s*)	TAF secretion rate	Estimated
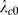	0	*ml/*(*cm* ^3^ *s*)	TAF consumption rate	Estimated
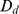	1.5×10^−14^	*m* ^2^/*s*	Drug diffusion coefficient	Estimated
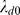	2.5×10^−7^	*ml/*(*cm* ^3^ *s*)	Drug consumption rate	Estimated
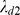	1×10^−8^	*ml/*(*cm* ^3^ *s*)	Drug decay rate	Estimated
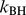	0.3×10^−3^	-	Branching hotpoints constant	Estimated
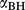	1.3	-	Branching hotpoints constant	Estimated
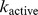	1.0	-	Rate of CVE addition by active cells	Estimated
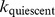	0.1	-	Rate of CVE addition by quiescent cells	Estimated
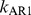	1.0	-	Vessel radius constant	Estimated
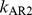	500.0	-	Vessel radius constant	Estimated
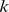	4.5×10^−15^	*cm* ^2^/*mmHg*−*sec*	Interstitium's hydraulic conductivity	Estimated
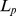	2.8×10^−9^	m/mm Hg-sec	Microvascular wall's hydraulic conductivity	[30]
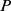	1.49×10^−9^	m/sec	Vascular permeability coefficient	Estimated
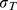	0.82	-	Average osmotic reflection coefficient	[30]
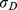	0.1	-	Average osmotic reflection coefficient	Estimated
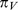	0.3546	mmHg	Osmotic pressure of plasma	Estimated
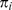	0.2667	mmHg	Osmotic pressure of interstitial fluid	Estimated
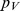	30	mmHg	Capillary/vascular pressure	Estimated
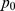	60	mmHg	Tumor pressure	Estimated
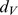	1.0	mol/m^3^	Interstitial drug concentration	Estimated
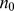	8.4	*mol/m* ^3^	Standard nutrient concentration	Estimated
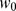	10.5	*mol/m* ^3^	Standard waste concentration	Estimated
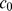	4.3×10^−4^	*kg/m* ^3^	Standard TAF concentration	[54]
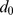	2.13	*mol/m* ^3^	Standard drug concentration	Estimated

### 3.1 Simulation Setup

The 3D virtual microenvironment for tumor simulation was set at 1 *cm* ^3^ divided by a discrete lattice with a grid of 200×200×200 points. Two separate lattices were constructed for tumor cells and endothelial cells, with the constraint that each grid point can contain only a single cell at each given timepoint. The starting oxygen (*O*_2_), carbon dioxide (*CO*_2_), TAF, and drug concentrations were assumed to be homogeneous 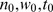 and *d*_0_ respectively. Dirichlet boundary conditions were set for each growth factor 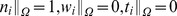, and 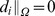 for computational purposes. Equations were normalized and then solved using Finite Difference Method (FDM). Initially at *t* = 0 there were five cancer cells were placed in the center of computational domain. As the tumor grew with time, the interstitial pressure, metabolite, and TAF concentrations were calculated accordingly for each grid point during simulation period. Cytotoxic drug was supplied via the vasculature at day 40th, and its relative concentration was calculated for each grid point over time. The following assumptions were made: 1) angiogenesis is induced by tumor cell quiescence; 2) tumor cells cycle once every 24 hours [38]; 3) cells reversibly enter quiescence when 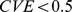; 4) cells irreversibly become necrotic when 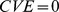; and 5) tumor pressure 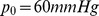 [27] and capillary pressure 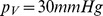 [39]. Tumor growth was simulated for 60 days (1 day equals to 33 steps or iterations) to cover the transition from avascular to vascular state. All simulations were performed on an Intel Core2 Quad 3.0 GHz CPU, 8 GB Memory desktop. The total simulation time was 12.68 hours.

### 3.2 Tumor Growth

**Figure 4:**
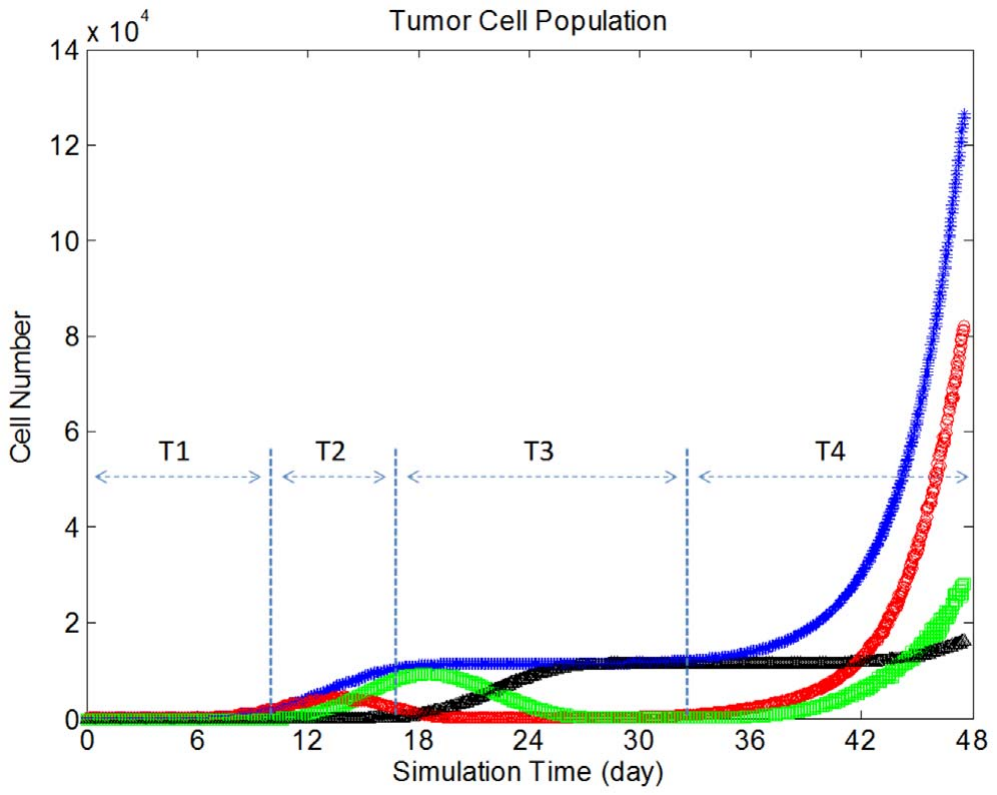
Tumor cell number over time. Blue: total cells; Red: active tumor cells; Green: quiescent tumor cells; Black: necrotic tumor cells.

**Figure 5:**
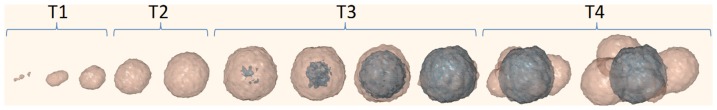
Tumor volume and morphology changes during progression including exponential growth, linear expansion, stasis, and secondary growth processes (T1–T4). Brown region: Viable cells; Black region: Necrotic cells.

The simulation results of tumor cell population changes with time are presented in Fig. 4 and Fig. 5. Fig. 4 shows the number of cells comprising the tumor and their relative metabolic state. The tumor growth process was characterized by four distinct stages: exponential growth (T1), linear expansion (T2), stasis (T3), and secondary growth (T4). Stages T1 and T2 represent the avascular state of tumor growth, in which tumor cells rapidly uptake oxygen and generate large amount of carbon dioxide during proliferation and proteins/DNA synthesis. As oxygen levels drop, the CVE is impaired and the cell cycle lengthens, resulting in the transition from T1 to T2. When the CVE drops below 0.5, tumor growth reaches a steady state characterized by an increase in cell quiescence. During stage T3, continued depletion of the CVE results in cell necrosis at the tumor core. The tumor cell number remains constant, however, the relative fraction of necrotic cells to quiescent cells increases over the course of several weeks. The initiation of angiogenesis at stage T4 generates a new supply of nutrients for further tumor growth, resulting in renewed cell proliferation. Fig. 5 shows tumor volume and morphology changes during this progression. Tumor volume changes were observed at stages T1, T2 and T4, whereas stage T3 was characterized by expansion of the necrotic core while the overall tumor volume remained constant.

### 3.3 Tumor Angiogenesis

**Figure 6:**
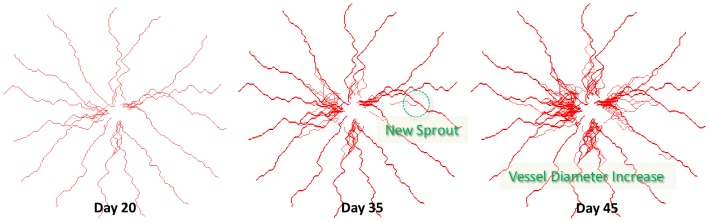
Angiogenic sprouting and vessel maturation during tumor growth. New vessel branches and sprouts occur at vessel branching hotpoints (VBHs) in response to changes in TAF concentration and interstitial pressure. These vessels grow towards hypoxic regions in order to provide independent blood supply. Vessel diameter as well as vessel density increases with time, shown here at timepoints. Note that the vessels are absent from tumor center, due the presence of a necrotic cell core.

Tumor-induced angiogenesis was studied using a refined lattice-based model in which endothelial cells proliferate up a TAF gradient at a rate determined by the local interstitial pressure. The baseline vessel growth rate was assumed to be 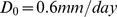 for normal tissue [40], which increased to 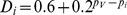 when vessels reached tumor tissue. Unlike studies in which angiogenesis was simulated only by tip cell division [41]–[43], here we also modeled vessel maturation and its influence on nutrient availability. Endothelial cell age was increased with each model iteration, such that vessel diameter and related average surface area per unit volume for mass transport [44] increased with time on a point-by-point basis (refer to Eqs. 22–24). Representative images of the tumor vasculature network at different stages is shown in Fig. 6.

### 3.4 Mass Transport

**Figure 7:**
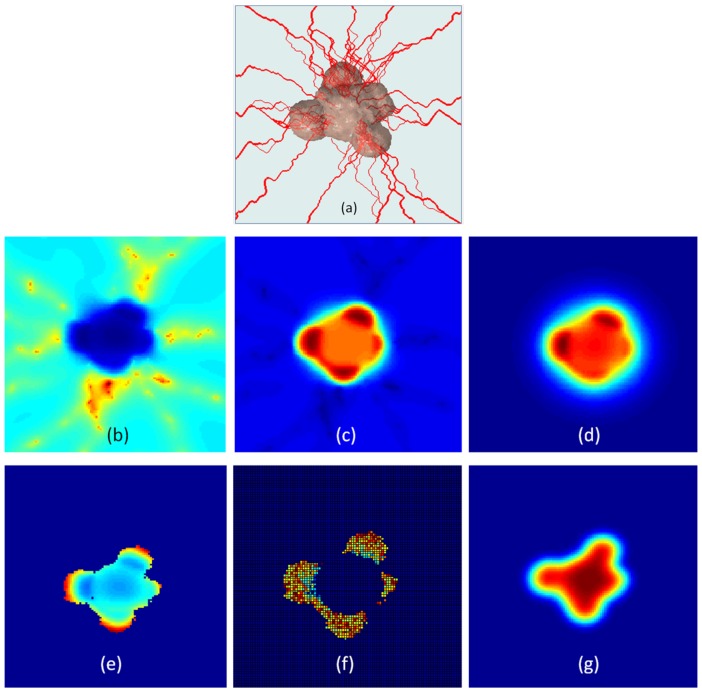
Influence of metabolite and growth factor distribution on tumor properties. (a) Vascularized 3D solid tumor morphology on Day 45; (b) Predicted oxygen distribution; (c) Predicted carbon dioxide distribution; (d) Predicted TAF distribution; (e) Cell activity distribution; (f) CVE distribution; (g) Interstitial pressure distribution; MDE distribution is similar to tumor pressure field. (Intensity: Red (high) → Blue (low)).

Various vascular abnormalities including shunts and tortuous, leaky vessels cause considerable blood and interstitial fluid infusion into tumor microenvironment [45], [46]. Meanwhile, deficient circulation and lymphatic drainage also add to this effect [47]. Our model predicts that the complex interplay between tumor interstitial pressure and the blood vessel distribution leads to spatial and temporal variations in metabolite and growth factor availability. Fig. 7 illustrates how this mass transport influences specific tumor features including tumor cell activity, CVE, and interstitial pressure. Tumor vessels appeared to preferentially localize at the periphery of the tumor (Fig. 7 (a)), resulting in high concentrations of oxygen near the tumor edge and little to no oxygen at the tumor center (Fig. 7 (b)). Carbon dioxide accumulation occurred throughout but was highest inside the tumor periphery (Fig. 7 (c)), likely due the presence of live cells combined with insufficient waste clearance. TAF was found to concentrate inside the tumor (Fig. 7 (d)), particularly in regions characterized by low oxygen content and high carbon dioxide content. Actively cycling cells appeared at the tumor periphery in regions of high oxygen influx and carbon dioxide clearance (Fig. 7 (e)). CVE was present in large portions of the tumor but absent within the necrotic core (Fig. 7 (f)). Individual CVE values varied by spatial position, likely due to local variations in metabolism, protein/DNA synthesis, ligand binding, etc. Tumor interstitial pressure was significantly higher than that of the surrounding tissue and was highest at the tumor core (Fig. 7 (g)). Literature [27], [48] suggests this phenomenon is due the fact that quickly dividing tumor cells are much more compact compared to normal cells, which pushes healthy tissue outward to form a boundary to trap interstitial fluid and pressure inside the tumor. Our model corroborates this, since here we considered both CTP and VTP, as well as probability of tumor cell movement or the direction of tumor growth. Despite modeling tumor growth coupled with angiogenesis, the other important processes in cancer progression such as cell detachment (from primary tumor mass), tissue invasion, intravasation, and circulation of tumor cells in blood vessels are not included in our model. Reader may refer to modeling papers by [49], [50] for modeling on the related issue.

### 3.5 Tumor Morphology

**Figure 8:**
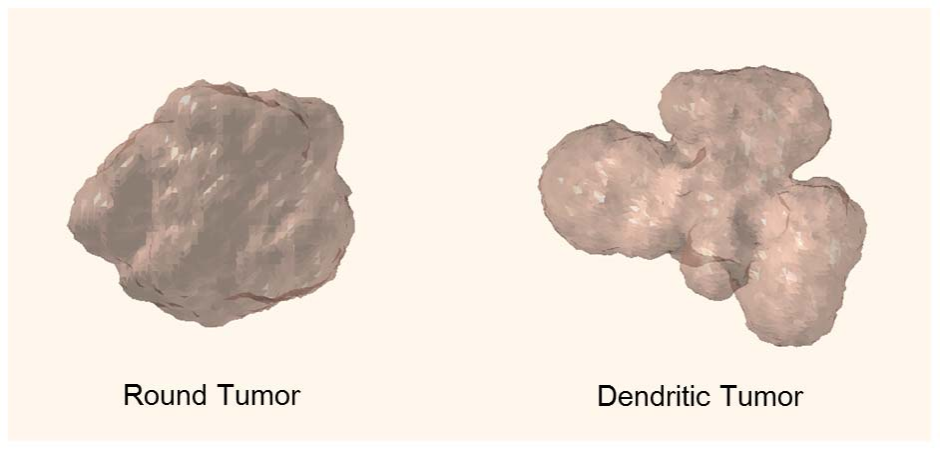
Predicted tumor morphology as a function of interstitial pressure. Low pressure tumors (40 mmHg) develop a rounded morphology (left) whereas high pressure tumors (60 mmHg) develop a dendritic morphology. Simulated time period: Day = 45.

In order to study tumor morphology with respect to interstitial pressure, we tested tumors of low (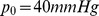) and high (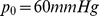) interstitial pressure while keeping the vascular pressure constant (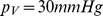). Computational results indicated that high-pressure tumors are more likely to develop dendritic structures compared to low-pressure tumors due to a stronger outwards interstitial fluid convection. This in turn reduces oxygen influx and waste clearance, favoring cell quiescence and/or necrosis at the tumor core. Thus we would expect well-vascularized cells at the tumor periphery to proliferate more rapidly, resulting in a dendritic structure, as shown in Fig. 8.

### 3.6 Tumor Growth Following 3D Vascular Inputs

**Figure 9:**
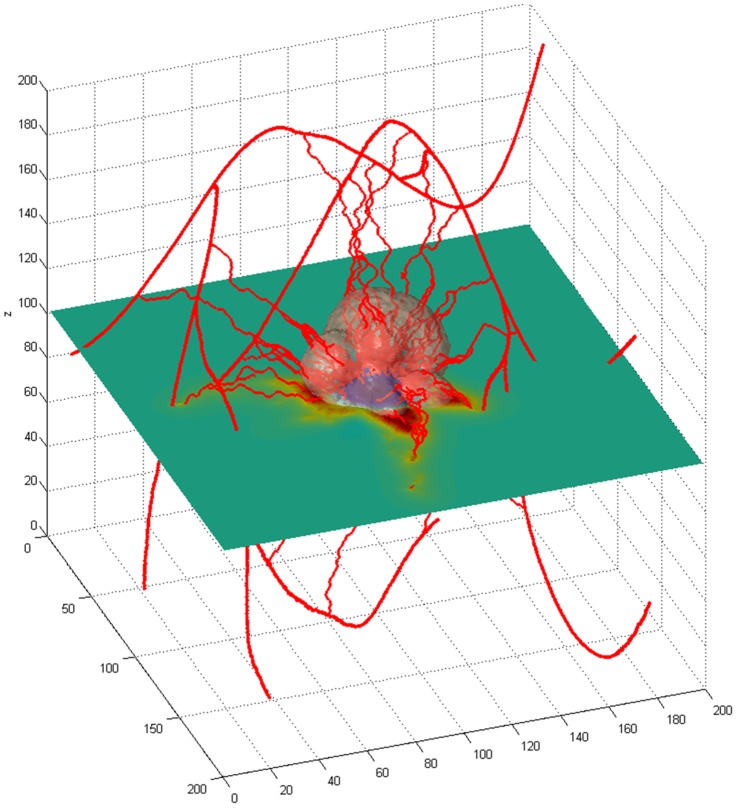
Solid tumor growth simulation with angiogenesis from a virtual 3D vasculature. The cross-sectional plane shows the nutrient availability within the simulation area.

By calibrating individual model parameters using MRI and intravital microscopy measurements, we were able to reconstruct 3D vasculature structures within the tumor growth simulation. In Fig. 9, we present one such simulation output. Multiple iterations of the simulation yielded angiogenic sprouting from the pre-existing vasculature at calculated VBH. Dendritic tumor growth was preferentially observed in areas of high vascularization and nutrient availability, as previously predicted. This example serves to illustrate a major strength of the model, that is, tumor growth and angiogenesis may be modeled using measured, site-specific vascular networks.

### 3.7 Cytotoxic chemotherapy

The toxicity of drugs targeted to tumor cells is determined by drug concentration and tumor cell activity, with higher concentrations of drug more likely to induce cell cycle transition from active to quiescent or necrotic (refer to Eq. 22). The intravascular drug concentration can be predicted using body level pharmacokinetics models, as introduced in [22], [51]. For simplicity, we treated the drug concentration as constant, similar to that observed with intravenous infusion. Nevertheless, time-varying plasma drug concentrations can be readily simulated. The baseline plasma drug concentration was set to *d*_0_ = 2.13 mol/m^3^, as commonly used for doxorubicin [52]. In our simulation, the concentration of drug in the tumor interstitium was influenced by the pressure difference within capillary blood and extracellular space, as well as vessel mass exchange surface per unit volume (as determined by vessel diameter and/or age). The cytotoxic drug was therefore more likely to diffuse into low pressure regions and unable reach inner tumor cells.

**Figure 10:**
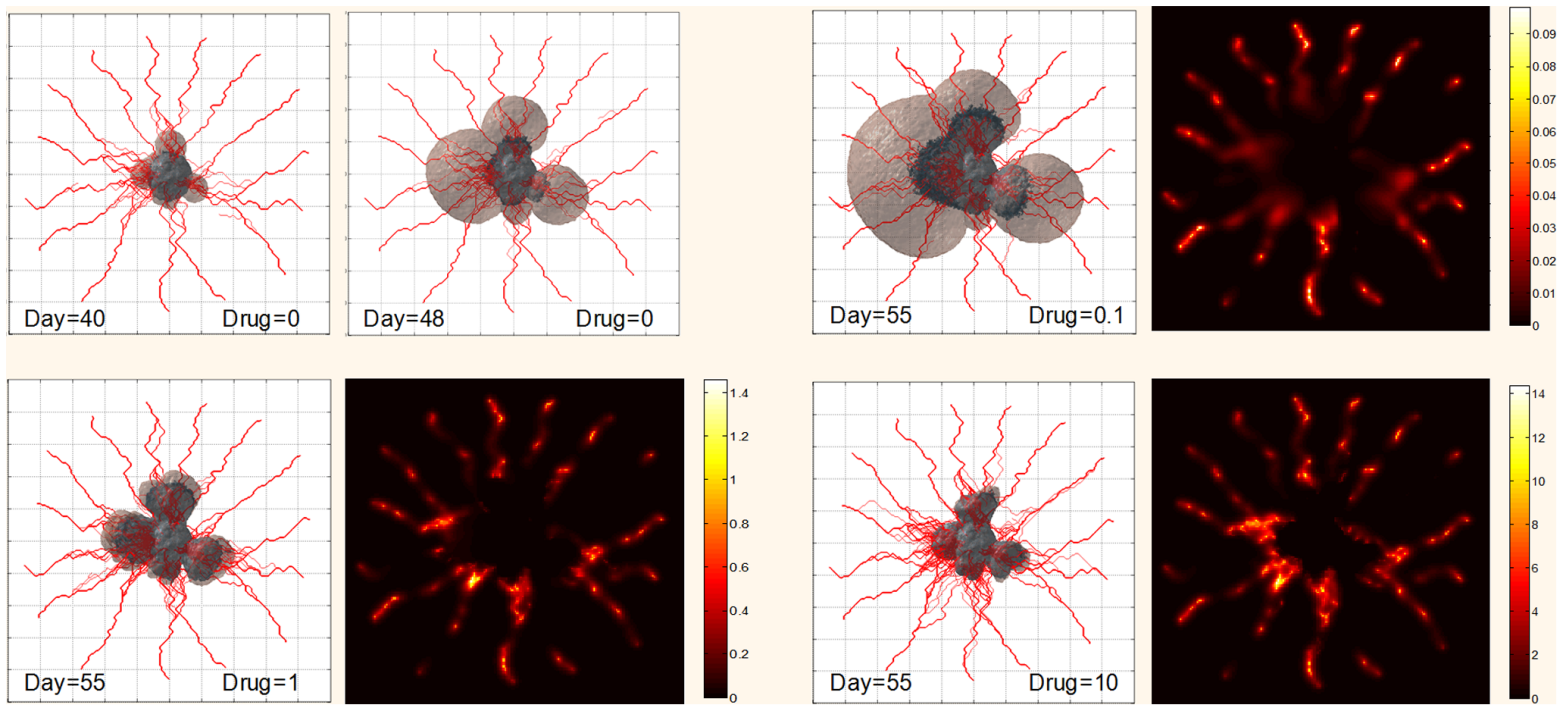
Tumor morphology and drug distribution following three different drug administration concentrations (0.1, 1, and 10 *mol/m^3^*), shown at normalized scale. In our model, drug is inserted at day 40. Two figures in the top left figures show tumor growth without drug. The rest figures show the effect of drug on tumor on the same day (55) with different drug concentrations (shown on the right figures with color bar). Brown region denotes living cells and black region indicates necrotic cells.

Cytotoxic therapy was simulated to start at day 40, concomitant with secondary tumor expansion. Fig. 10 shows representative illustrations of drug distribution and tumor size after treatment. A low concentration of drug (0.1 *mol/m* ^3^) was found to produce little growth suppression, resulting in expansion of both the viable cells and necrotic core. Increasing the drug concentration to 1 *mol/m* ^3^ resulted in slowed proliferation of the active cells and a more compact tumor morphology. Large doses of drug (10 *mol/m* ^3^) produced significant cell apoptosis.

### 3.8 Parameter sensitivity analysis

We performed parameter sensitivity analysis to examine the robustness of the system of our model, that is to evaluate if varying key parameters may affect the results. We varied the parameters *ρ_n_*_0_ and *λ_n_*_0_ in Eq. (6), *ρ_w_*_0_ and *λ_w_*_0_ in Eq. (9), *ρ_c_*_0_ in Eq. (15), and *λ_d_*_0_ and *λ_d_*_2_ in Eq. (21) by reducing and increasing their values 0.1%, 1%, and 10% from their default values listed in Table 1. The results are presented in Table 2, where we compared the average value of the main variables of the system model, that is nutrient, waste, TAF, and drug. The results in Table 2 are the percentage changes of the values of varying parameters with respect to the values using default parameters, arranged in order of “nutrient/waste/TAF/drug”, taken at simulation day 60. For example, in the first row and first column for results of *ρ_n_*_0_ reduced to −10%, the percentage change of nutrient is 0.18, waste is 0, TAF is 0, and drug is 2.7. As we observe in Table 2, the average value of the variables are quite robust with regard to changes of the parameters. We can see that the most sensitive variable to changes is TAF, which is found to have large sensitivity when varying *λ_n_*_0_.

**Table 2:** Results of the parameter sensitivity analysis by varying key parameters of nutrient (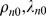), waste (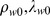), TAF (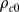), and drug (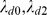).

	−10%	−1%	−0.1%	+0.1%	+1%	+10%
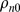	0.18/0/0/2.7	0.11/0.01/0.37/5.41	0.07/0.01/1.87/2.7	0.08/0.01/0.75/5.41	0.07/0.01/0.75/5.41	0.01/0.02/1.87/8.11
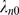	0.25/0.14/25.37/2.7	0.52/0.1/20.52/13.51	0.47/0.13/27.99/5.41	0.09/0.08/18.66/8.11	0.52/0.1/21.27/13.51	0.35/0.12/22.76/2.7
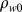	0.12/0/3.73/5.41	0.07/0/0.75/5.41	0/0/0.37/0	0.07/0.01/0.37/5.41	0.02/0.01/0.75/2.7	0.01/0.02/2.61/2.7
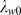	0.06/0.01/0.37/5.41	0.01/0.01/0.37/0	0.11/0.02/1.49/5.41	0.05/0.01/0.37/2.7	0.02/0/0.37/2.7	0.15/0.01/1.87/8.11
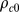	0.07/0.01/4.48/2.7	0.11/0.01/0.75/8.11	0.04/0.01/0.75/2.7	0.09/0.01/0.37/5.41	0/0.01/0.75/0	0.05/0.01/6.72/2.7
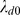	0.09/0.01/0.75/5.41	0.13/0/0.75/8.11	0.09/0/1.12/8.11	0.08/0/1.12/8.11	0.08/0/1.12/5.41	0.04/0/1.12/2.7
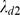	0.06/0.01/0.75/2.7	0.15/0.01/0.37/8.11	0.17/0.01/0.37/10.81	0.16/0.01/0.37/10.81	0.16/0.01/0.75/10.81	0.16/0.01/0.37/10.81

The results are the percentage changes of varied parameters with respect to default values presented in Table 1, arranged in order of “nutrient/waste/TAF/drug”.

## Discussion

Here we proposed a multi-scale tumor growth and angiogenesis model for chemotherapy evaluation. At tissue level, we calculated the tumor interstitial pressure based on a GPM which incorporates pressure induced by tumor cell contact and vascular perfusion. The GPM model can adaptively calculate tumor pressure during tumor growth from an avascular to vascular state with relatively low computational costs. The model is not limited to tumors of rounded morphology and can be applied to tumors with dendritic morphology and unclear boundaries. Incorporating tumor pressure-induced interstitial fluid convection, we built a series of mass conservation partial differential equations to model oxygen, carbon dioxide, and TAF distributions at intratumoral level. With this information, detailed and comprehensive mechanisms of tumor cell proliferation and endothelial cell angiogenesis were proposed at cellular level in order to provide high-resolution predictions for single drug regimen. For future work, we will extend the model where an expression for anti-angiogenic drug can be included. Anti-angiogenic drugs target immature vessels and may cause endothelial cell apoptosis. Systematic computational simulations conducted in this paper illustrate the advantages of our model. Multiple features of early stage tumor progression, including tumor cell expansion, morphology changes, and cell phenotype transitions were simulated in our model. Furthermore, since the 3D tumor morphology and growth patterns may vary by tumor origin and/or metastatic site, we incorporated tissue-specific variables, including tissue density, growth factor diffusion rates, and vasculature structure. Being comprised of different functional modules that are easily modified and refined, the model has the potential to incorporate additional details, including cell signaling pathways, drug molecule properties, and local perfusion characteristics. With such additional inputs and calibration, the model may be customized for specific applications. We envision that the proposed model and its future advances can serve as a valuable predictive platform for cancer drug discovery, screening, and testing.
